# Therapeutic Agents Targeting the Nrf2 Signaling Pathway to Combat Oxidative Stress and Intestinal Inflammation in Veterinary and Translational Medicine

**DOI:** 10.3390/vetsci13010025

**Published:** 2025-12-25

**Authors:** Muhammad Zahoor Khan, Shuhuan Li, Abd Ullah, Yan Li, Mohammed Abohashrh, Fuad M. Alzahrani, Khalid J. Alzahrani, Khalaf F. Alsharif, Changfa Wang, Qingshan Ma

**Affiliations:** 1 College of Agriculture and Biology, Liaocheng University, Liaocheng 252000, China; zahoorkhan@lcu.edu.cn (M.Z.K.);; 2 Department of Basic Medical Sciences, College of Applied Medical Sciences, King Khalid University, Abha 61421, Saudi Arabia; 3 Department of Clinical Laboratories Sciences, College of Applied Medical Sciences, Taif University, Taif 21944, Saudi Arabia

**Keywords:** Nrf2, NF-κB, intestinal health, oxidative stress, inflammation, antioxidants, gut barrier function, livestock

## Abstract

This review synthesizes research on nuclear factor erythroid 2-related factor 2 (Nrf2) in intestinal health across human, livestock, and mouse models. The Nrf2 signaling pathway serves as a master regulator of cellular antioxidant defenses and a key therapeutic target for intestinal inflammatory disorders, including ulcerative colitis and Crohn’s disease. The interplay between oxidative stress, Nrf2 signaling, and NF-κB inflammatory cascades represents a critical axis in the pathogenesis and resolution of intestinal inflammation. Under normal physiological conditions, Nrf2 remains sequestered in the cytoplasm by Kelch-like ECH-associated protein 1 (Keap1), which facilitates its ubiquitination and proteasomal degradation. However, during oxidative stress, reactive oxygen species (ROS) and electrophilic compounds modify critical cysteine residues on Keap1, disrupting the Keap1-Nrf2 interaction and enabling Nrf2 nuclear translocation. Once in the nucleus, Nrf2 binds to antioxidant response elements (ARE) in the promoter regions of genes encoding phase II detoxifying enzymes and antioxidant proteins, including heme oxygenase-1 (HO-1), NAD(P)H quinone oxidoreductase 1 (NQO1), and glutamate-cysteine ligase. This comprehensive review synthesizes current evidence demonstrating that activation of Nrf2 signaling confers protection against intestinal inflammation through multiple interconnected mechanisms: suppression of NF-κB-mediated pro-inflammatory cascades, enhancement of cellular antioxidant capacity, restoration of intestinal barrier integrity, modulation of immune cell function, and favorable alteration of gut microbiota composition. We systematically examine a diverse array of therapeutic agents targeting Nrf2 signaling, including bioactive peptides, natural polyphenols, flavonoids, terpenoids, alkaloids, polysaccharides, probiotics, and synthetic compounds. The mechanistic insights and therapeutic evidence presented underscore the translational potential of Nrf2 pathway modulation as a multi-targeted strategy for managing intestinal inflammatory conditions and restoring mucosal homeostasis.

## Simple Summary

The gut plays a critical role in animal and human health, with inflammatory conditions causing significant welfare and economic concerns. This review examines how the Nrf2 signaling pathway protects the intestine from damage caused by oxidative stress and inflammation. We explore various natural compounds (polyphenols, terpenoids, alkaloids), probiotics, and nutritional interventions that activate Nrf2 to enhance antioxidant defenses, suppress inflammatory responses, strengthen intestinal barriers, and beneficially modulate gut microbiota. The evidence demonstrates that Nrf2 activation represents a promising multi-targeted therapeutic strategy for managing intestinal inflammatory disorders in both clinical and livestock settings.

## 1. Introduction

Inflammatory bowel diseases (IBD), encompassing ulcerative colitis (UC) and Crohn’s disease (CD), represent a group of chronic, relapsing inflammatory disorders of the gastrointestinal tract that afflict millions of individuals worldwide [1,2,3]. These conditions impose a substantial burden on healthcare systems and significantly diminish patients’ quality of life through persistent symptoms including abdominal pain, diarrhea, rectal bleeding, weight loss, and systemic complications [4,5,6,7]. The pathogenesis of IBD is multifactorial, involving complex interactions between genetic susceptibility, environmental triggers, intestinal microbiota dysbiosis, dysregulated immune responses, and impaired mucosal barrier function [8,9]. A hallmark feature of IBD is the generation of excessive reactive oxygen species (ROS) and sustained oxidative stress within the intestinal mucosa, which perpetuates tissue damage, amplifies inflammatory cascades, and compromises epithelial barrier integrity [10,11,12].

The nuclear factor erythroid 2-related factor 2 (Nrf2) serves as the master transcriptional regulator orchestrating cellular antioxidant and cytoprotective responses [13,14,15]. Under homeostatic conditions, Nrf2 activity is tightly regulated by Kelch-like ECH-associated protein 1 (Keap1), which promotes Nrf2 degradation to maintain low basal expression of antioxidant genes [16]. Upon exposure to oxidative stress or electrophilic compounds, Nrf2 stabilizes and translocates to the nucleus, activating transcription of phase II detoxifying enzymes, antioxidant proteins (heme oxygenase-1, superoxide dismutase, catalase, glutathione peroxidase), glutathione biosynthesis enzymes, and numerous other cytoprotective mediators [17,18,19,20]. In intestinal epithelium, Nrf2 activation enhances barrier function, promotes epithelial regeneration, modulates immune responses, and influences microbiota composition, thereby protecting against oxidative damage and inflammation [21,22,23].

The therapeutic potential of Nrf2 pathway modulation in intestinal health has gained considerable attention in recent years [24,25]. Accumulating evidence from preclinical studies demonstrates that pharmacological activation of Nrf2 signaling through diverse natural products, synthetic compounds, and dietary interventions can ameliorate experimental colitis, reduce intestinal inflammation, restore barrier integrity, and modulate gut microbiota composition [26,27]. These beneficial effects are mediated through multiple interconnected mechanisms, including enhanced antioxidant capacity, suppression of pro-inflammatory signaling cascades [particularly the nuclear factor kappa B (NF-κB) pathway], inhibition of inflammasome activation, modulation of immune cell function, promotion of intestinal stem cell activity, and favorable alteration of microbial ecology [28,29]. The cross-talk between Nrf2 and NF-κB pathways represents a particularly important regulatory axis, as activation of Nrf2 can suppress NF-κB-driven inflammatory gene expression through multiple mechanisms, including direct protein–protein interactions, competition for transcriptional co-activators, and indirect effects mediated by Nrf2-induced antioxidant proteins that reduce ROS-dependent NF-κB activation [28,29,30].

These mechanisms underpin their ability to improve gut morphology, modulate microbiota, enhance immune function, and alleviate stress-induced intestinal damage, thereby improving growth performance [31]. Despite these promising physiological and performance-related benefits, current evidence remains limited and heterogeneous across different species and experimental conditions. While their potential in animal husbandry is promising, future research must address gaps in comparative efficacy, synergistic effects, and long-term safety to enable practical applications [32,33,34]. This comprehensive review aims to synthesize current understanding of Nrf2 signaling in intestinal health, with particular emphasis on the molecular mechanisms underlying the protective effects of Nrf2 activation against intestinal inflammation. We systematically examine the intricate interplay between oxidative stress, Nrf2 signaling, and NF-κB inflammatory cascades in the pathogenesis and resolution of intestinal inflammation. With a focus on translational applications across veterinary and clinical settings, we provide a detailed analysis of diverse therapeutic agents that target Nrf2 signaling to ameliorate intestinal inflammatory conditions, including natural bioactive compounds, probiotics, dietary interventions, and synthetic molecules.

## 2. Literature Search Strategy

A targeted literature search was conducted using PubMed, google scholar, Web of Science, and Scopus databases with the following key terms: Nrf2 signaling, intestinal health, inflammatory bowel disease, intestinal inflammation, colitis, ulcerative colitis, and oxidative stress. Boolean operators (AND, OR) were used to combine search terms. Studies involving livestock, in vitro models, and mouse models were included. Reference lists of relevant reviews were also screened for additional studies. To capture recent advancements in Nrf2 signaling as a therapeutic target for intestinal health, the search was limited to articles published between 2019 and 2025. Only peer-reviewed original research articles and reviews published in English and indexed in science citation index (SCI) journals were considered for inclusion. Conference abstracts, non-peer-reviewed publications, and non-English articles were excluded.

## 3. The Interplay of Oxidative Stress, Nrf2 Signaling, and NF-κB Pathway in Intestinal Inflammation

### 3.1. Oxidative Stress in IBD Pathophysiology

Inflammatory bowel diseases exhibit pathologically increased reactive oxygen species (ROS) generation through multiple enzymatic sources, including nicotinamide adenine dinucleotide phosphate (NADPH) oxidases (NOX1, NOX2), mitochondrial electron transport chain dysfunction, xanthine oxidase, and myeloperoxidase released from activated neutrophils [35,36]. This oxidative burden overwhelms endogenous antioxidant defenses, manifesting as elevated lipid peroxidation products (malondialdehyde, 4-hydroxynonenal), protein carbonylation, oxidized glutathione ratios, and DNA oxidation markers (8-hydroxy-2′-deoxyguanosine) [37,38]. The resulting redox dysregulation perpetuates mucosal injury through multiple mechanisms. Oxidative modification of membrane phospholipids compromises epithelial barrier integrity through tight junction protein degradation (occludin, claudins, ZO-1) and cytoskeletal disruption, facilitating paracellular permeability and bacterial translocation [39,40,41]. ROS function as critical second messengers activating redox-sensitive transcription factors, particularly NF-κB, through IκB kinase phosphorylation and subsequent IκBα degradation, thereby amplifying pro-inflammatory gene expression and cytokine production (TNF-α, IL-1β, IL-6) [42].

### 3.2. Nrf2-Keap1 Regulatory Architecture

The Keap1-Nrf2 axis functions as a molecular rheostat governing cellular redox homeostasis and cytoprotective responses [13,14,15]. Under homeostatic conditions, Keap1 homodimers sequester Nrf2 in the cytoplasm through BTB (broad complex, tramtrack, bric-à-brac) domain-mediated association with the Cullin 3-Rbx1 E3 ubiquitin ligase complex [43]. This molecular architecture maintains constitutive Nrf2 ubiquitination and rapid proteasomal turnover, ensuring low basal expression of ARE-dependent genes [44]. Keap1 contains multiple reactive cysteine residues (Cys151, Cys273, Cys288) functioning as electrophile sensors that undergo post-translational modifications (oxidation, alkylation, nitrosylation) upon encountering oxidative or electrophilic stressors [45]. These modifications disrupt the hinge-and-latch mechanism between Keap1’s double glycine repeat (DGR) domain and Nrf2’s Neh2 domain, stabilizing Nrf2 and promoting nuclear accumulation [46]. Nuclear Nrf2 heterodimerizes with small Maf proteins (MafF, MafG, MafK) and binds antioxidant response elements (ARE) in promoter regions, recruiting transcriptional co-activators (CBP/p300) to orchestrate expression of >200 cytoprotective genes encoding phase II detoxifying enzymes (NQO1, GSTs, UGTs), antioxidant proteins (HO-1, GCLC/M, thioredoxin, peroxiredoxins), and metabolic regulators [46,47].

### 3.3. Nrf2-NF-κB Crosstalk in Intestinal Inflammation

The Nrf2 and NF-κB signaling pathways engage in extensive bidirectional crosstalk that determines the balance between cytoprotection and inflammation in intestinal tissues [25,28,48]. ROS serve as obligate second messengers driving NF-κB activation through oxidative modification of pathway components, including IκB kinase complex phosphorylation and IκBα degradation [49]. Conversely, Nrf2-mediated induction of antioxidant enzymes attenuates ROS-dependent NF-κB activation, creating a negative feedback loop [50]. Direct protein–protein interactions provide additional regulatory complexity: Keap1 associates with IKKβ modulating its activity, while Nrf2 physically interacts with p65/RelA, either sequestering it in the cytoplasm or competing for limiting transcriptional co-activators (CBP/p300) in the nucleus [51]. Temporal dynamics distinguish these pathways: NF-κB exhibits rapid, transient activation kinetics (minutes) suited for immediate inflammatory responses, whereas Nrf2 demonstrates delayed but sustained activation (hours) appropriate for establishing adaptive cytoprotection [52].

**Figure 1:**
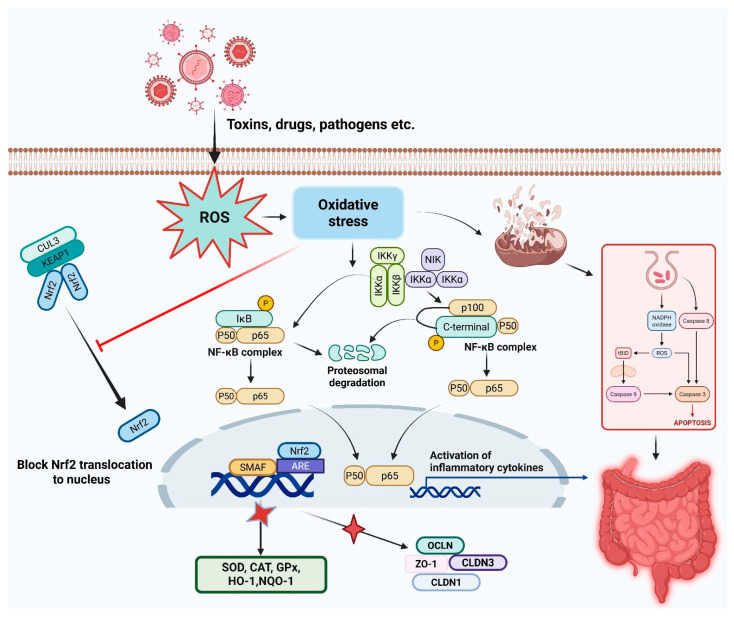
Schematic representation of how various environmental, dietary, and pathological agents induce oxidative stress and trigger intestinal diseases including inflammatory bowel diseases. The figure illustrates the pathways leading from oxidative stress inducers to cellular damage, barrier dysfunction, and chronic inflammation in the gastrointestinal tract.

In experimental colitis models, Nrf2^−^/^−^ mice display heightened disease severity with exacerbated histological damage, increased NF-κB activity, and elevated inflammatory cytokine expression [48,53]. Pharmacological Nrf2 activation confers protection through multi-modal mechanisms: enhanced antioxidant capacity, attenuated pro-inflammatory signaling, preserved epithelial barrier function via tight junction protein upregulation, favorable modulation of immune cell phenotypes (M2 macrophage polarization, regulatory T cell expansion), and beneficial microbiota compositional shifts [53,54,55]. These integrated effects collectively restore intestinal homeostasis and promote mucosal healing in inflammatory conditions. The mechanisms by which oxidative stress and various environmental, dietary, and pathological agents trigger intestinal diseases are summarized in Figure 1.

## 4. Therapeutic Agents Targeting Nrf2 Signaling for Intestinal Health

### 4.1. Natural Phenolic Compounds

The extensive body of research on natural phenolic compounds as Nrf2 activators underscores their significant therapeutic potential for intestinal inflammatory disorders. The structural diversity of these compounds—from simple phenolic acids to complex flavonoids and stilbenoids—is matched by a consistent ability to enhance antioxidant defenses and suppress pro-inflammatory pathways. Importantly, these compounds have demonstrated efficacy across multiple experimental platforms, including rodent colitis models, porcine and avian livestock systems, and human intestinal cell lines, supporting their translational relevance for both agricultural applications and human inflammatory bowel disease (IBD) therapeutics. The key compounds, their sources, mechanisms, and outcomes discussed in this section are systematically summarized in Table 1.

#### 4.1.1. Stilbenoids

Resveratrol demonstrates multifaceted Nrf2-mediated intestinal protection beyond antioxidant effects. In DSS models, it activates Nrf2/HO-1 signaling, enhancing transepithelial electrical resistance and upregulating tight junction proteins ZO-1 and Occludin [54]. Resveratrol simultaneously suppresses NF-κB p65 translocation while activating Nrf2/HO-1, addressing both oxidative and inflammatory injury components in zearalenone-induced damage [48]. Network pharmacology confirms its effects operate primarily through Nrf2-dependent oxidative stress modulation [54]. Notably, resveratrol counteracts ferroptosis through Nrf2/FTH1/GPX4 axis activation in exercise-induced intestinal damage [56], positioning it as a prototype for understanding multi-pathway protection through upstream Nrf2 activation. Collectively, stilbenoids, particularly resveratrol, represent well-characterized Nrf2 activators with robust preclinical evidence supporting their development as therapeutic agents for oxidative stress-related intestinal pathologies in both veterinary and human medicine.

#### 4.1.2. Phenolic Acids

Phenolic acids demonstrate mechanistic precision in Nrf2 activation while revealing gut microbiota’s critical role in therapeutic efficacy. Caffeic acid dietary administration in piglets enhanced growth and gut health, with in vitro H_2_O_2_ protection completely abrogated by Nrf2 inhibitor ML385, establishing unequivocal pathway dependency [21]. Ellagic acid exhibits bidirectional microbiota interactions, with intestinal biotransformation into bioavailable urolithin metabolites that activate Nrf2 while inhibiting NF-κB [57]. Ellagic acid also modulates microbiome composition, reducing oxidative stress via Nrf2/ROS/ NOD-like receptor pyrin domain-containing protein 3 (NLRP3) axis [58], and mitigates paraquat-induced stress through Nrf2 translocation and HO-1/NQO1 upregulation [59].

Paralleling these mechanistic insights, chlorogenic acid exemplifies dual-pathway regulation, inhibiting TLR4/NF-κB while activating Nrf2-mediated antioxidant responses. In piglets, supplementation elevated systemic antioxidant capacity and modulated ileal microbiota [49]. Its protective effects extend to the gut–brain axis, protecting against sleep deprivation-induced cognitive deficits through Nrf2/PPAR activation while enhancing barrier function [60]. The phenolic acid family demonstrates consistent dose-dependent Nrf2/HO-1 activation: ferulic and quinic acids reduce inflammation in acetic acid-induced colitis [61], while coumaric and syringic acids alleviate colitis with significant TNF-α and IL-1β reductions [62]. Tannic acid engages the p62-Keap1-Nrf2 pathway while inhibiting TLR4-NF-κB-NLRP3 signaling against ETEC K88-induced diarrhea [63], suggesting autophagy-dependent Nrf2 activation mechanisms. Taken together, phenolic acids constitute a versatile class of Nrf2 activators whose microbiota-mediated biotransformation and dual antioxidant-anti-inflammatory actions establish them as promising candidates for translational development across agricultural and clinical settings.

#### 4.1.3. Coumarins

Coumarins combine Nrf2-mediated protection with direct antimicrobial activity. For example, Esculetin exhibits antibacterial activity against multidrug-resistant *Salmonella typhimurium* through downregulation of the T3SS-1 virulence system, preventing bacterial invasion of intestinal cells, and in infected mice, esculetin ameliorated cecal injury and corrected microbial dysbiosis through Nrf2 pathway activation [64]. This dual antimicrobial and cytoprotective mechanism is complemented by scopoletin, which demonstrates the characteristic dual-pathway mechanism by suppressing NF-κB while activating Nrf2 to upregulate HO-1 and NQO1, with restoration of tight junction proteins confirming barrier-protective effects [65]. The dual antimicrobial-cytoprotective properties of coumarins position them as particularly valuable therapeutic candidates in the context of rising antimicrobial resistance. Their ability to simultaneously target bacterial virulence mechanisms and reinforce host intestinal defenses through Nrf2 activation represents a novel therapeutic strategy applicable to both food animal production—where reducing antibiotic usage is a priority—and human gastrointestinal infections complicated by dysbiosis. Thus, coumarins represent a distinct subclass of phenolic compounds whose combined antimicrobial and Nrf2-activating properties offer unique advantages for managing infectious enteritis across species.

#### 4.1.4. Flavonoids

Flavonoids represent the most extensively studied polyphenol class for intestinal Nrf2 modulation. Morin reverses deoxynivalenol-induced damage through direct Keap1 inhibition, activating Nrf2 and enhancing intestinal stem cell proliferation [24]. Morin hydrate functions as a dual Nrf2 and PPARγ agonist with stable molecular interactions confirmed by docking studies [66]. Complementing this pharmacological approach, Apigenin pretreatment alleviates ischemia/reperfusion injury by upregulating Nrf2, HO-1, and tight junction proteins, with Nrf2 knockdown abolishing protection [26].

Building on these findings, naringenin activates Nrf2 components (HO-1, NQO1) while suppressing NF-κB, reducing TNF-α and IL-1β in UC models [67]. Naringin modulates stress and inflammation through coordinated TLR4/p38 MAPK/NF-κB inhibition and Nrf2 activation, affecting tight junctions, mucin 2, and permeability markers [68]. Quercetin provides compelling Nrf2-dependent barrier protection, with effects requiring Nrf2 expression [69]. Dose-dependent activation reverses DON-induced claudin-4 reduction, completely blocked by brusatol [70]. Quercetin also protects against combined zearalenone and lipopolysaccharide (LPS) toxicity through upregulation of HO-1, SOD2, and NQO1 [71], and against radiation-induced injury through ROS curtailment [72].

Extending beyond conventional flavonoids, Genistein reverses H_2_O_2_-induced Nrf2 suppression, protecting barrier integrity [46], and alleviates IBD through dual mechanisms: enriching *Akkermansia muciniphila* while activating GPR30-Nrf2 axis to suppress NLRP3 inflammasome [73]. Paralleling this multifaceted approach, baicalin attenuates barrier damage through AMPK/Nrf2 modulation, with AMPK knockdown abolishing protection [74]. In addition, baicalein suppresses ferroptosis by upregulating GPX4 and FTH1 via Nrf2 while modulating gut microbiota [75]. Moreover, rutin improves barrier integrity through Nrf2/Keap1 activation with concurrent cecal microbiota modulation [76], and taxifolin demonstrates consistent protection across multiple stress models [40,77]. Expanding this therapeutic repertoire, additional flavonoids with documented Nrf2-mediated protection include pectolinarigenin [78], wogonin [79], and Eucommia ulmoides flavonoids [50]. Overall, flavonoids constitute the most extensively validated class of natural Nrf2 activators, with mechanistically diverse compounds offering multiple therapeutic entry points for intestinal inflammatory disorders across veterinary and human medical applications.

#### 4.1.5. Specialized Polyphenols

Curcumin represents the most extensively characterized Nrf2 activator. Dietary curcumin mitigates AFB1 toxicity by upregulating Nrf2, HO-1, and NQO-1 systemically [43]. Mechanistically, it activates SIRT1/NRF2 while inhibiting TLR4, reducing NLRP3-mediated pyroptosis [80]. Notably, nanoparticle formulations (Theracurmin) by targeting NRF2 signaling, significantly attenuate DSS-induced colitis while inducing regulatory T cells [81]. Complementing these findings, epigallocatechin gallate (EGCG) alleviates cisplatin-induced injury through Nrf2/Keap1 activation with microbiota and autophagy modulation, with ML385 completely reversing protection [54]. Extending this mechanistic diversity, carthamin yellow activates Nrf2/GPX4 axis to counter ferroptosis in UC, with effects abolished in Nrf2-knockout mice and with ML385 treatment [82]. Moreover, hydroxytyrosol concurrently activates PI3K/Akt-Nrf2 signaling and promotes mitophagy, with disruption of either pathway compromising protection [83], revealing synergistic mechanisms whereby Nrf2 clears ROS through antioxidant upregulation while mitophagy eliminates damaged mitochondria. These findings demonstrate that specialized polyphenols exemplify the successful translation of Nrf2-targeting compounds from preclinical models to clinical application, with advanced formulation strategies and emerging mechanistic insights continuing to expand their therapeutic potential.

**Table 1:** Natural Phenolic Compounds Targeting Nrf2 Signaling for Intestinal Health.

Model Category	Compound	Category	Model System	Key Pathways	Ref.
Rodent Models	Resveratrol	Stilbenoid	DSS-induced colitis mice; ZEA-injured mice; High-intensity exercise mice	Nrf2/HO-1, NF-κB; Nrf2/FTH1/GPX4	[6,11,48,56]
	Resveratrol Analog C33	Stilbenoid	Mouse colitis model; Nrf2-KO mice	Nrf2	[84]
	Sericic Acid	Triterpenoid	DSS-induced colitis mice	NF-κB, Nrf2	[85]
	Dehydrocostus Lactone	Sesquiterpene	DSS-induced colitis	NF-κB, Keap1/Nrf2	[86]
	SLRF	Sesquiterpene	DSS-induced UC	Nrf2-Hmox-1, NF-κB/MAPK	[87]
	Epoxymicheliolide	Sesquiterpene	DSS-induced colitis	TAK1-NF-κB, Keap1/Nrf2	[88]
	Gingerenone A	Phenolic	DSS-induced colitis mice	Nrf2-Gpx4	[89]
	Carnosic Acid	Diterpene	DSS-induced colitis mice	Microbiota → Nrf2	[90]
	Sesamin	Lignan	DSS-induced murine colitis	AKT/ERK → Nrf2	[91]
	Ellagic Acid	Phenolic Acid	DSS-induced colitis mice	Nrf2, NF-κB, ROS/ NLRP3	[57,58]
	Chlorogenic Acid	Phenolic Acid	Sleep-deprived mice	Nrf2/PPAR	[60]
	Ferulic/Quinic Acid	Phenolic Acid	Acetic acid-induced colitis rats	Nrf2/HO-1	[61]
	Coumaric/Syringic Acid	Phenolic Acid	Colitis model	Nrf2/HO-1	[62]
	Esculetin	Coumarin	Salmonella-infected mice	Nrf2	[64]
	Scopoletin	Coumarin	DSS-induced UC mice	Nrf2/HO-1/NQO1, NF-κB	[65]
	Umbelliferone	Coumarin	Acetic acid-induced UC rats	TLR4/NF-κB, SIRT1/PPARγ	[92]
	Apigenin	Flavonoid	Rat I/R model	Nrf2/HO-1	[26]
	Naringenin	Flavonoid	DSS-induced UC mice	Nrf2/HO-1/NQO1, NF-κB	[67]
	Naringin	Flavonoid	LPS-challenged mice	Nrf2, TLR4/p38 MAPK/NF-κB	[68]
	Quercetin	Flavonoid	Radiation-injured mice	Nrf2	[72]
	Diosmin	Flavonoid	DSS-induced colitis mice	Nrf2, NF-κB	[93]
	Curcumin (Theracurmin)	Polyphenol	DSS-induced colitis mice	Nrf2/regulatory T cells	[81]
	EGCG	Polyphenol	Cisplatin-injured rats	Nrf2/Keap1	[54]
	Carthamin Yellow	Polyphenol	DSS-induced colitis mice	Nrf2/GPX4	[82]
	Moringin	Polyphenol	DSS-induced colitis mice	Nrf2/NF-κB, PI3K/AKT/mTOR	[94]
	Geniposide	Polyphenol	DSS-induced colitis mice	Nrf2/ARE, NF-κB	[72]
	Grape Seed Anthocyanins	Polyphenol	DSS-induced colitis mice	Nrf2, TLR4/NF-κB	[34]
	Loganic Acid	Iridoid	DSS-induced colitis mice	TLR4/NF-κB, SIRT1/Nrf2	[95]
Cell Line Models	Resveratrol	Stilbenoid	IPEC-J2 cells; H_2_O_2_-induced	PI3K/Akt/Nrf2	[96]
	Resveratrol	Stilbenoid	DSS-induced IEC barrier dysfunction	Nrf2/HO-1	[97]
	Resveratrol	Stilbenoid	TNF-α-challenged Caco-2	Nrf2/IL-1β/IL-11	[98]
	Sesamin	Lignan	Caco-2 cells	AKT/ERK → Nrf2	[94]
	Epoxymicheliolide	Sesquiterpene	Macrophages	TAK1-NF-κB, Keap1/Nrf2	[91]
	Apigenin	Flavonoid	H/R cells	Nrf2/HO-1	[26]
	Genistein	Flavonoid	H_2_O_2_-induced IPEC-J2 cells	Nrf2; GPR30-Nrf2	[46,47,73]
	Baicalin	Flavonoid	H_2_O_2_-induced IPEC-J2 cells	AMPK/Nrf2	[74,75]
	Taxifolin	Flavonoid	Diquat/DON-induced IPEC-J2	Nrf2	[40,77]
	Astaxanthin	Polyphenol	AFB1-exposed IPEC-J2 cells	Nrf2/HO-1/NQO1/SOD2	[99]
	Gardenin A	Polyphenol	Alcohol-damaged HepG2/Caco2	AMPK/Nrf2	[39]
	Carthamin Yellow	Polyphenol	LPS-stimulated Caco-2	Nrf2/GPX4	[82]
	Moringin	Polyphenol	LPS-stimulated Caco-2	Nrf2/NF-κB	[97]
	Geniposide	Polyphenol	LPS-stimulated Caco-2	Nrf2/ARE, NF-κB	[72]
Domestic Animals	Pterostilbene	Stilbenoid	Livestock models	Nrf2/HO-1, NF-κB	[6,11]
	Caffeic Acid	Phenolic Acid	Weaned piglets; IPEC-J2	Nrf2	[21]
	Chlorogenic Acid	Phenolic Acid	Weaned piglets	Nrf2, TLR4/NF-κB	[49]
	Tannic Acid	Phenolic Acid	ETEC K88-challenged piglets	p62-Keap1-Nrf2, TLR4-NF-κB	[63]
	Morin	Flavonoid	DON-induced damage (livestock)	Keap1/Nrf2	[24,25]
	Quercetin	Flavonoid	DON-challenged piglets	Nrf2	[70,71]
	Rutin	Flavonoid	Weaned piglets	Nrf2/Keap1	[76]
	EUF	Flavonoid	DON-challenged piglets	Nrf2/Keap1	[50]
	Curcumin	Polyphenol	AFB1-exposed broilers	Nrf2/HO-1/NQO-1	[43]
	Hydroxytyrosol	Polyphenol	Diquat-induced pig model	PI3K/Akt-Nrf2, Mitophagy	[83]
	Oat Bran Polyphenols	Polyphenol	Livestock applications	ROS/Akt/Nrf2	[100]

### 4.2. Bioactive Nutrients and Microbial Agents

**Figure 2:**
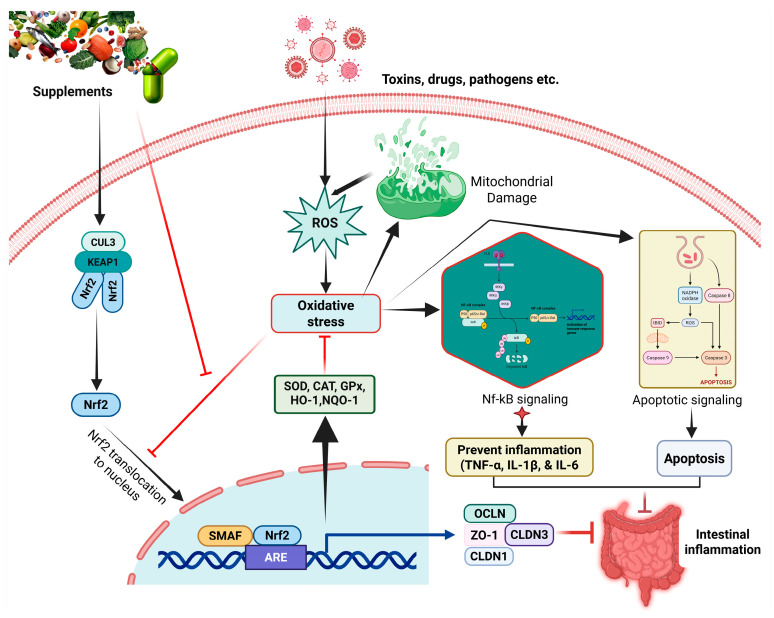
Nrf2 signaling pathway as a therapeutic target for intestinal inflammation. The figure depicts the Keap1-Nrf2 regulatory axis, showing how Nrf2 activation leads to transcription of antioxidant response element (ARE)-dependent genes including HO-1, NQO1, and glutathione synthesis enzymes, ultimately providing protection against oxidative stress and inflammation in intestinal epithelial cells.

Beyond phenolic compounds, a wide array of bioactive nutrients, microbial agents, and their metabolites also exert protective effects on intestinal health primarily through the modulation of the Nrf2 pathway. These agents, including polysaccharides, peptides, probiotics, and short-chain fatty acids, often function through unique mechanisms involving gut microbiota-mediated biotransformation. Critically, these compounds are particularly amenable to dietary supplementation strategies in both livestock production and human nutrition, facilitating their practical implementation across diverse settings. The evidence for these diverse Nrf2-targeting nutrients and microbial agents is consolidated in Table 2, and their mechanisms are shown in Figure 2.

#### 4.2.1. Polysaccharides

Complex polysaccharides demonstrate Nrf2-activating mechanisms involving gut microbiota-mediated biotransformation and prebiotic effects. For example, brown algae polysaccharides (BAPs) alleviate oxidative stress through Nrf2/ARE activation, increasing nuclear Nrf2 translocation and upregulating HO-1, SOD, and CAT [101]. Epimedium polysaccharide alleviates UC by activating Keap1/Nrf2 while promoting protective autophagy via AMPK/mTOR, with Nrf2-dependent efficacy demonstrated in knockout mice. Efficacy was demonstrated to be Nrf2-dependent using knockout mice, establishing the hierarchy of pathway activation [102]. Similarly, *Artemisia annua* polysaccharide improves growth performance in E. coli-challenged broilers while enhancing antioxidant capacity through Nrf2 activation and TLR4/MyD88/NF-κB inhibition [103]. Furthermore, Mussel polysaccharide activates Nrf2-Keap1 pathway with HO-1 and antioxidant enzyme upregulation, protecting against cyclophosphamide-induced oxidative injury [104].

Extending beyond direct Nrf2 activation, *Lycium barbarum* polysaccharides activate Nrf2 signaling while enriching beneficial microbiota (*Ruminococcaceae*, *Lactobacillus*, *Akkermansia*) and enhancing short-chain fatty acid production [105]. Paralleling this integrated approach, polysaccharide suppresses NF-κB and NLRP3 inflammasome while activating Nrf2/HO-1 and mediating anti-ferroptosis effects [106,107]. Additional mechanistically characterized compounds include *Melientha longistaminea* polysaccharide [108], *Floccularia luteovirens* polysaccharide [109,110], *Artemisia capillaris* polysaccharide [111], yeast β-glucan (activating both Nrf2 and AHR) [112], and *Camellia oleifera* polysaccharide [113] as shown in Table 2. Accordingly, polysaccharides represent multifunctional Nrf2 activators whose combined antioxidant, anti-inflammatory, and prebiotic properties position them as ideal candidates for dietary-based intestinal health interventions across species.

#### 4.2.2. Peptides and Amino Acids

Bioactive peptides provide intestinal protection through Nrf2-mediated antioxidant responses combined with immunomodulation. Peptide-induced Nrf2 activation upregulates antioxidant enzymes, reducing ROS and subsequently inhibiting ROS-sensitive NF-κB [17]. For example, activates Keap1-Nrf2 pathway, with LC-MS identifying four specific bioactive sequences [114]. Complementing these findings, rice protein peptides have been found to ctivate Keap1-Nrf2 signaling while modulating gut microbiota, increasing *Firmicutes*/*Bacteroidetes* ratio and promoting beneficial bacteria [115].

In addition to bioactive peptides, individual amino acids also demonstrate Nrf2-activating capacity beyond nutritional roles. For example, proline upregulates Nrf2/HO-1, suppressing NF-κB p65 phosphorylation to alleviate hibernation-induced barrier dysfunction [116]. While, taurine produces dose-dependent improvements in antioxidant status through enhanced Nrf2, HO-1, GPX1, and SOD1 expression, with optimal doses (0.3–0.4%) favorably modulating gut microbiota [117]. In addition, L-theanine has been shown to promote Nrf2 nuclear translocation while decreasing Keap1, with ML385 completely abolishing protection [118]. Extending this mechanistic diversity, glutamine prevents 5-FU-induced mucositis by inhibiting TLR4/NF-κB while activating Nrf2/HO-1 and increasing mTOR, with concurrent microbial diversity enhancement [119]. Furthermore, betaine activates Nrf2/Keap1 while inhibiting TLR4-NF-κB/MAPK, with effects transmissible through maternal supplementation [120], demonstrating transgenerational protective mechanisms. From a practical standpoint, amino acids and bioactive peptides constitute an immediately translatable class of Nrf2 activators, with established clinical and agricultural applications that can be enhanced through mechanistic understanding of their Nrf2-dependent protective actions.

#### 4.2.3. Fatty Acids and Lipids

Dietary lipids demonstrate Nrf2-activating properties contributing to intestinal health benefits. α-Linolenic acid activates NRF2 signaling dose-dependently, with 600 mg/kg identified as optimal [121]. Similarly, Deer oil suppresses NF-κB phosphorylation while upregulating Nrf2/HO-1, with parallel microbiota modulation increasing *Odoribacter*, *Blautia*, and short-chain fatty acids [30]. Moreover, Coix seed oil activates Keap1/Nrf2 in kidney while reinforcing intestinal barrier integrity through tight junction upregulation [41]. Expanding this therapeutic repertoire, α-Lipoic acid activates Keap1-Nrf2 while inhibiting both ferroptosis and endoplasmic reticulum stress [122]. Furthermore, icosapent ethyl activates SIRT1/Nrf2/HO-1 and PI3K/Akt signaling while suppressing NF-κB, with benefits abolished by SIRT1 inhibition [123]. Hence, dietary lipids represent an accessible class of Nrf2 activators whose protective intestinal effects complement their established systemic health benefits, supporting integrated dietary strategies for gut-systemic health optimization.

#### 4.2.4. Organic Acids

Simple organic acids demonstrate Nrf2-activating capacity useful in feed additives and functional foods. Glyceryl lactate combined with lactic acid activates Nrf2 while modulating cecal microbiota to increase SCFA-producing bacteria, proving most effective in combination [124]. Building on this synergistic principle, encapsulated benzoic acid outperforms unencapsulated forms in suppressing TLR4/NF-κB and activating NRF2, with NRF2 inhibition abolishing benefits [108]. Complementing these findings, itaconic acid activates Keap1/Nrf2/HO-1 while reversing gut dysbiosis induced by perfluorooctanoic acid (PFOA) exposure, reducing harmful bacteria while promoting *Lactobacillus* [121]. Notably, organic acids offer practical, cost-effective Nrf2 activation strategies particularly suited to large-scale livestock production, with potential extension to human functional food development.

#### 4.2.5. Probiotics

Probiotic organisms activate Nrf2 through microbial metabolite production, pattern recognition receptor engagement, or direct host cell interaction. Bacteroides thetaiotaomicron increases tryptophan metabolites including indole, enhancing barrier integrity and reducing inflammation via AHR-Nrf2 signaling [22]. Building on this framework, *Lactobacillus acidophilus* activates p62-Keap1-Nrf2 pathway while increasing *Akkermansia* and restoring short-chain fatty acids [31]. Similarly, *Clostridium dalinum* inhibits NF-κB while activating Keap1-Nrf2-ARE, with metabolomics identifying elevated anti-inflammatory metabolites and butanoate metabolism activation [32]. Expanding this mechanistic repertoire, *Lacticaseibacillus paracasei* CCFM1222 inhibits TLR4/MyD88/NF-κB while activating Nrf2, elevating antioxidant enzymes and shifting microbiota [29]. Moreover, *Clostridium butyricum* enhances intestinal health by activating Nrf2 and suppressing NF-κB while improving digestive enzyme activity [105], whereas *Bacillus amyloliquefaciens* mediates protection through Keap1/Nrf2 signaling, increasing Nrf2 while decreasing Keap1 [45]. As such, probiotics exemplify the successful integration of Nrf2-targeting strategies with microbiome modulation, offering established therapeutic platforms for continued development in both veterinary and human intestinal health applications.

#### 4.2.6. Microbial Metabolites

Microbiota-derived metabolites represent an emerging therapeutic class mediating many probiotic and prebiotic effects. Indole-3-lactic acid, produced by *Lactiplantibacillus plantarum* DPUL-S164 from tryptophan metabolism, strengthens intestinal barrier via coordinated AhR and Nrf2 activation, with both dependent on AhR expression [125]. Paralleling these findings, sodium butyrate alleviates DSS-induced colitis by activating COX-2/Nrf2/HO-1 while suppressing NF-κB/NLRP3 inflammasome and promoting mitophagy via Pink1/Parkin [126]. Furthermore, butyric acid upregulates Nrf2 in vitro, with fecal microbiota transplantation from treated mice replicating therapeutic effects and Nrf2 activation [127], demonstrating that microbiota-derived butyrate is sufficient to activate host Nrf2 with functional consequences. Consequently, microbial metabolites constitute an emerging class of Nrf2 activators that bridge dietary, microbiome, and pharmaceutical intervention strategies, offering precise molecular tools for modulating intestinal oxidative stress responses.

**Table 2:** Bioactive nutrients and microbial agents targeting Nrf2 for intestinal health.

Model Category	Compound	Category	Model System	Key Pathways	Ref.
Rodent Models	Spermidine	Polyamine	Experimental IBD; colitis models	AhR-Nrf2, AhR-STAT3	[128]
	Carbocisteine	Mucoregulator	Acetic acid-induced UC rats	Nrf2/HO-1, NF-κB	[129]
	Myristicin	Phenylpropanoid	Acetic acid-induced UC rats	ERS, Nrf2/HO-1, NF-κB	[130]
	Sulforaphane	Isothiocyanate	DSS-induced UC mice	Nrf2, STAT3	[131]
	MLS Polysaccharide	Polysaccharide	CTX-induced immunosuppression mice	NF-κB, Nrf2	[108]
	FLP1 Polysaccharide	Polysaccharide	Mouse immunosuppression model	MAPK, Nrf2/Keap1	[109,110]
	APS Polysaccharide	Polysaccharide	ANIT-induced cholestasis mice	Nrf2 (via butyric acid)	[111]
	Mussel Polysaccharide	Polysaccharide	Cyclophosphamide-injured mice	Nrf2-Keap1/HO-1	[104]
	EPS Polysaccharide	Polysaccharide	DSS-induced UC mice; Nrf2 KO	Keap1/Nrf2, AMPK/mTOR	[102]
	LBP Polysaccharide	Polysaccharide	DSS-induced chronic UC mice	Nrf2	[105]
	Wheat Peptide	Peptide	DSS-induced colitis mice	Keap1-Nrf2	[114]
	Glutamine	Amino Acid	5-FU-induced mucositis mice	Nrf2/HO-1, TLR4/NF-κB	[119]
	Deer Oil	Lipid	DSS-induced UC mice	Nrf2/HO-1, NF-κB	[30]
	Coix Seed Oil	Lipid	Hyperuricemia mice	Keap1/Nrf2, PI3K/AKT/mTOR	[41]
	α-Lipoic Acid	Fatty Acid	DSS-induced UC mice	Keap1-Nrf2	[122]
	Icosapent Ethyl	Fatty Acid	Acetic acid-induced UC rats	SIRT1/Nrf2/HO-1, NF-κB	[123]
	TDCA	Bile Acid	Diquat-induced mice	Nrf2	[132]
	*B. thetaiotaomicron*	Probiotic	Cold-stressed mice	AHR-Nrf2	[22]
	*L. acidophilus*	Probiotic	Salmonella-infected mice	p62-Keap1-Nrf2	[31]
	*L. paracasei* CCFM1222	Probiotic	Murine colitis model	Nrf2, TLR4/MyD88/NF-κB	[29]
	L. casei + VIP	Probiotic + Peptide	DSS-induced UC mice	Nrf2, NF-κB	[133]
	Sodium Butyrate	Metabolite	DSS-induced murine colitis	Nrf2/HO-1, NF-κB/NLRP3	[126]
Cell Line Models	Encapsulated Benzoic Acid	Organic Acid	LPS-challenged IPEC-J2	NRF2, TLR4/NF-κB	[36]
	*Clostridium dalinum*	Probiotic	Caco-2 cells	Keap1-Nrf2-ARE, NF-κB	[32]
	*B. amyloliquefaciens*	Probiotic	LPS-challenged Caco-2	Keap1/Nrf2	[45]
	Indole-3-lactic Acid	Metabolite	LPS-damaged HT-29 cells	AhR-Nrf2	[125]
	α-Lipoic Acid	Fatty Acid	Erastin-induced cells	Keap1-Nrf2	[122]
Domestic Animals	Selenomethionine	Selenium	DON-induced damage; ISC (piglets)	Keap1/Nrf2	[134]
	BAO (Benzoic + EO)	Organic Acid/EO	LPS-challenged weaned piglets	Nrf2, TLR4/NF-κB/MAPK	[127]
	BAPs	Polysaccharide	Diquat-challenged piglets	Nrf2/ARE	[101]
	AAP Polysaccharide	Polysaccharide	*E. coli*-challenged broilers	Nrf2, TLR4/MyD88/NF-κB	[103]
	Proline	Amino Acid	Turtles’ post-hibernation	Nrf2/HO-1, NF-κB	[116]
	Taurine	Amino Acid	Early-weaned piglets	Nrf2/HO-1/GPX1/SOD1	[117]
	L-theanine	Amino Acid	Weaned piglets; IPEC-J2	Nrf2	[118]
	Betaine	Amino Acid	Sows/offspring piglets	Nrf2/Keap1, TLR4-NF-κB	[120]
	α-Linolenic Acid	Fatty Acid	Broilers (42-day)	NRF2/HO-1	[35]
	Itaconic Acid	Organic Acid	perfluorooctanoic acid (PFOA)-exposed laying hens	Keap1/Nrf2/HO-1, NF-κB	[121]
	LA/GL	Organic Acid	28-day piglet study	Nrf2	[124]
	*C. butyricum*	Probiotic	Broilers	Nrf2, NF-κB	[135]
	*B. amyloliquefaciens*	Probiotic	Broilers	Keap1/Nrf2	[45]

### 4.3. Terpenoids, Alkaloids, Plant Extracts and Traditional Medicines in Intestinal Health

The therapeutic repertoire of Nrf2 activators extends further into structurally complex terpenoids, potent alkaloids, and multi-component plant extracts and traditional formulas. These compounds often exhibit high-affinity interactions with the Keap1-Nrf2 complex and engage in sophisticated multi-pathway regulation. Notably, many of these agents derive from traditional medicine systems with centuries of empirical validation in treating gastrointestinal disorders, providing a strong rationale for their contemporary mechanistic investigation and clinical development. The broad spectrum of these plant-derived therapeutics and their documented efficacy in experimental models of intestinal inflammation are cataloged in Table 3.

#### 4.3.1. Terpenoids

Terpenoids demonstrate Nrf2-activating mechanisms often involving direct protein-target interactions amenable to structural characterization. Cardamonin functions through hierarchical AhR activation that subsequently promotes Nrf2 nuclear translocation and NLRP3 inflammasome inhibition, with both CH223191 (AhR inhibitor) and ML385 (Nrf2 inhibitor) abolishing anti-inflammatory effects [33]. Moreover, efficacy extends to heat stress conditions, activating Nrf2 components (Nrf2, HO-1, NQO1) while modulating gut microbiota [136]. Building upon these hierarchical activation mechanisms, neferine competitively binds Keap1 at Cys-288, promoting Nrf2 nuclear translocation and activating Nrf2/FPN and Nrf2/xCT/GPX4 axes. This dual-axis activation inhibits ferroptosis in severe acute pancreatitis, with effects reversed by ML385 and erastin [27].

Extending beyond single-pathway modulation, α-mangostin activates Nrf2/HO-1 while suppressing NF-κB/NLRP3/caspase-1, reducing oxidative stress and promoting autophagy. Treatment remodeled gut microbiota, increasing *Ligilactobacillus* and *Muribaculum* that correlated with elevated propionic and butyric acid [137]. Similarly, sclareol activates Nrf2 while inhibiting NF-κB/MLCK pathway, preserving tight junctions and reducing inflammation and bacterial translocation [138]. Furthermore, limonin demonstrates direct Nrf2/ARE interaction via molecular docking, with ML385 reversing protection against NSAID-induced injury [139]. Complementing these findings, oleanolic acid 28-O-β-D-glucopyranoside (OAG) alleviates UC by inhibiting ferroptosis through Nrf2/x-CT/GPX4. Multi-omics analysis confirmed OAG directly binds and modulates GPX4, NRF2, HO-1, and x-CT [140]. Additional terpenoids include panaxadiol (inhibiting MAPK/NF-κB while activating AMPK/NRF2/NQO1) [141], columbianadin (potentiating Nrf2 while suppressing TLR4/NF-κB) [99,142], and melianodiol (activating Nrf2 while suppressing JAK/STAT and NF-κB) [143]. Together, these data indicate that terpenoids represent a structurally diverse class of Nrf2 activators whose well-defined molecular interactions facilitate rational drug design and clinical translation for intestinal inflammatory disorders.

#### 4.3.2. Alkaloids

Alkaloids demonstrate potent Nrf2-activating capacity with well-characterized molecular mechanisms. Berberine produces marked Nfe2l2 upregulation with enhanced SOD, CAT, GPx, and GR activities in experimental colitis [144]. In ETEC-infected piglets, berberine downregulates TLR4/MyD88/NF-κB while upregulating Nrf2, optimizing gut microbiota by increasing beneficial bacteria [145]. Notably, berberine also regulates gut microbiota-associated tryptophan metabolites through AhR activation, restoring gut barrier function through this microbiota-metabolite-receptor axis [146]. Complementing these findings, sinomenine activates Nrf2/HO-1 while suppressing NF-κB to reduce TNF-α, IL-6, and IL-1β. It modulates cell death processes (necroptosis, pyroptosis) and remodels gut microbiota by correcting *Firmicutes* and *Bacteroidetes* imbalances [147]. Similarly, cepharanthine activates AMPK-α1/AKT/GSK-3β signaling, driving NRF2-dependent antioxidant genes (HO-1, NQO-1) while inhibiting MAPK and NF-κB p65. Effects were entirely abrogated in NRF2-knockout mice [148]. Isoquinoline alkaloids from Macleaya cordata co-regulate TLR4/MyD88/NF-κB and Nrf2 pathways, restoring gut microbiota and barrier integrity [149]. Oxyberberine, a microbiota-derived berberine metabolite with superior efficacy, activates Keap1/Nrf2 by promoting Nrf2 nuclear translocation while suppressing NF-κB through IκBα phosphorylation inhibition [150]. Additional alkaloids include dictamnine (activating Nrf2-Gpx4 to inhibit ferroptosis) [151], corynoline (with ML385-abolished protection) [152], and cinnamaldehyde (activating PI3K/Akt/Nrf2 to restore barrier integrity) [47]. Based on these findings, alkaloids constitute potent, clinically validated Nrf2 activators with established safety profiles and regulatory precedent, positioning them as leading candidates for continued therapeutic development in intestinal inflammatory disorders.

#### 4.3.3. Plant Extracts

Complex plant extracts demonstrate therapeutic effects potentially through synergistic multi-target engagement or enhanced bioavailability. For instance, *Hericium erinaceus* extract activates Nrf2 and upregulates HO-1 while enhancing microbiome diversity, providing gastroprotection against ethanol-induced and acetic acid-induced injury [153]. Similarly, *Rosa roxburghii* fermentation broths activate Nrf2/HO-1/NQO1 through Nrf2 nuclear translocation, attenuating pulmonary fibrosis while modulating gut microbiota. Correlation analysis linked microbial changes (reduced Proteus, increased *Ileibacterium* and *Dubosiella*) to oxidative stress inhibition [154]. In a comparable manner, Glycine tabacina ethanol extract suppresses NF-κB and MAPK/JNK while activating Nrf2, coordinately reducing pro-inflammatory cytokines, matrix metalloproteinases, and oxidative stress [28]. Furthermore, Viola yedoensis Makino inhibits NF-κB/NLRP3 while activating Nrf2/MAPK, reducing mitochondrial damage and apoptosis while modulating microbiota [155]. Correspondingly, *Forsythia suspensa* extract activates Nrf2-NLRP3 coordination, with Nrf2 silencing abolishing anti-pyroptotic effects. Metabolomic analysis revealed reversal of colitis-associated dysfunction in glutathione metabolism, aminoacyl-tRNA biosynthesis, and linoleic acid metabolism [156]. Additional plant extracts with characterized Nrf2-mediated protection include Aurantii Fructus extract (activating Nrf2/HO-1 while suppressing NF-κB and restoring microbiota) [157], Mesua Assamica extract (suppressing NF-κB/STAT3 while activating HO-1/Nrf2/SIRT1) [158], apple polyphenols (exhibiting Nrf2-dependent barrier protection reduced by ML385) [159], grape seed anthocyanins encapsulated in chitosan-phytic acid gel (inhibiting TLR4/NF-κB while activating Nrf2) [34], and maggot extracts (increasing Nrf2 while preventing Keap1-mediated degradation, abolished by ML385) [160]. Therefore, plant extracts represent pragmatic Nrf2-targeting interventions whose multi-component nature may confer therapeutic advantages through synergistic pathway modulation, supporting their development as functional foods and nutraceuticals for intestinal health.

#### 4.3.4. Traditional Herbal Formulas

Classical herbal formulations represent empirically validated multi-component therapeutics converging on Nrf2 modulation. Lizhong decoction (LZD) alleviates UC by inhibiting ferroptosis through Nrf2 activation, upregulating SLC7A11 and GPX4 while suppressing oxidative stress and iron overload [161]. Building upon these observations, licorice and its principal active component glycyrrhetinic acid activate Nrf2/PINK1 to regulate mitophagy, reducing inflammation and oxidative stress while repairing mitochondrial damage and enhancing barrier proteins. Gene silencing abolished protective effects [162,163]. Similarly, Gegen Qinlian decoction (GQ) activates Nrf2/ARE to combat oxidative stress, with puerarin, berberine, and liquiritin identified as primary drivers [164].

Certain formulations achieve effects through microbiota-mediated mechanisms converging on Nrf2 signaling. In contrast to the direct pathway activation described above, Yinchen Linggui Zhugan decoction modifies gut microbiota to enrich SCFA-producing bacteria and elevate butyric acid, which subsequently activates hepatic SIRT1/Nrf2 to upregulate HO-1 and NQO1 [136]. Ginseng root extract (GRE) suppresses MAPK/NF-κB while activating p62-Nrf2-Keap1 axis to enhance antioxidant defense and mitigate mitochondrial dysfunction. GRE also induces cytoprotective autophagy via Akt-mTOR pathway [107]. Ultimately, traditional herbal formulas represent a rich source of validated Nrf2-targeting therapeutics whose integration of empirical clinical evidence with contemporary molecular mechanistic understanding offers a robust foundation for evidence-based application in modern intestinal disease management.

**Table 3:** Terpenoids, alkaloids, plant extracts and traditional medicines.

Model Category	Compound	Category	Model System	Key Pathways	Ref.
Rodent Models	Ruscogenin	Saponin	TNBS-induced Crohn’s-like colitis	Nrf2/NQO1/HO-1	[165]
	Bryostatin-1	Macrolide	Intestinal I/R injury mice	Nrf2/HO-1	[166]
	Curculigoside	Glycoside	Murine UC models	Keap1/Nrf2 → autophagy	[167]
	Cannabidiol	Cannabinoid	DSS-induced colitis	Nrf2/HO-1, NF-κB, TGF-β	[168]
	Mushroom Extract (AP + FV)	Extract	DSS-induced UC with liver injury	TLR4/NF-κB, Keap1/Nrf2	[169]
	Andrographolide	Diterpenoid	DSS-induced mice	Nrf2/HO-1	[170]
	Coix Seed	Functional Food	DSS-induced UC mice	Src/JNK MAPK, Nrf2/PPARγ	[171]
	Neferine	Terpenoid	Severe acute pancreatitis mice	Nrf2/FPN, Nrf2/xCT/GPX4	[27]
	α-Mangostin	Terpenoid	Alcohol-induced gastric ulcers rats	Nrf2/HO-1, NF-κB/NLRP3	[172]
	Sclareol	Terpenoid	TNBS-induced CD mice	Nrf2, NF-κB/MLCK	[138]
	Limonin	Terpenoid	Indomethacin-induced injury rats	Nrf2/ARE	[139]
	Zingerone	Terpenoid	HFD-induced duodenal injury rats	Nrf2, NF-κB	[173]
	Melatonin	Terpenoid-like	BPA-exposed colon	SIRT1/PGC-1α/Nrf2	[174]
	Ginsenoside Rg1	Terpenoid	DSS-induced UC mice	Nrf-2/HO-1/NF-κB	[151]
	Berberine	Alkaloid	ETEC-infected piglets	Nrf2, TLR4/MyD88/NF-κB	[145]
	Oxyberberine	Alkaloid	TNBS-induced colitis rats	Keap1/Nrf2, NF-κB	[150]
	Sinomenine	Alkaloid	DSS-induced UC rats	Nrf2/HO-1, NF-κB	[147]
	Cepharanthine	Alkaloid	Experimental colitis; Nrf2-KO	AMPK/AKT/GSK-3β → NRF2	[148]
	Columbianadin	Alkaloid	DSS-induced UC rats	Nrf2, TLR4/NF-κB	[142]
	Hernandezine	Alkaloid	DSS-induced colitis	AMPK/NRF2	[175]
	Oleracein E	Alkaloid	TNBS-induced rats	Nrf2/HO-1	[40]
	GTE	Plant Extract	DSS colitis mice	Nrf2, NF-κB, MAPK/JNK	[28]
	*Hericium erinaceus* Extract	Plant Extract	Ethanol/acetic acid-injured rats	Nrf2/HO-1	[153]
	RRFBs	Plant Extract	Bleomycin-induced fibrosis mice	Nrf2/HO-1/NQO1	[154]
	SP Extract	Plant Extract	DSS-induced UC mice	Nrf2/Keap1/HO-1/NQO1	[176]
	PHE	Plant Extract	DSS-induced colitis	KEAP1 → Nrf2	[177]
	Lizhong Decoction	Herbal Formula	DSS-induced UC mice	Nrf2/SLC7A11/GPX4	[161]
	Licorice	Herbal Formula	DSS-induced UC mice	Nrf2/PINK1	[162,163]
	Gegen Qinlian Decoction	Herbal Formula	DSS-induced UC rats	Nrf2/ARE	[164]
	OAG	Triterpene	TNBS-induced UC rats	Nrf2/x-CT/GPX4	[140]
	Lithospermic Acid	Plant Compound	Murine colitis	Nrf2 + NF-κB inhibition	[178]
Cell Line Models	Ruscogenin	Saponin	LPS-stimulated organoids	Nrf2/NQO1/HO-1	[165]
	Bryostatin-1	Macrolide	Cellular models	Nrf2/HO-1	[166]
	Curculigoside	Glycoside	Organoid, cellular UC models	Keap1/Nrf2 → autophagy	[167]
	Andrographolide	Diterpenoid	LPS-induced cells	Nrf2/HO-1	[170]
	α-Mangostin	Terpenoid	GSE-1/RAW264.7 cells	Nrf2/HO-1, NF-κB/NLRP3	[172]
	Sclareol	Terpenoid	TNF-α-induced organoids	Nrf2, NF-κB/MLCK	[138]
	Cinnamaldehyde	Terpenoid	P. gingivalis-infected IEC-6	PI3K/Akt/Nrf2	[47]
	Oleracein E	Alkaloid	LPS-stimulated cells	Nrf2/HO-1	[40]
	Lizhong Decoction	Herbal Formula	RSL3-induced Caco-2	Nrf2/SLC7A11/GPX4	[161]
	Licorice	Herbal Formula	LPS-induced Caco2	Nrf2/PINK1	[163]
	Gegen Qinlian Decoction	Herbal Formula	TNF-α-stimulated Caco-2	Nrf2/ARE	[164]
	Lithospermic Acid	Plant Compound	NCM460 cells	Nrf2 + NF-κB inhibition	[178]
Domestic Animals	Cardamonin	Terpenoid	LPS-challenged piglets; Heat-stressed chickens	AhR/Nrf2/NLRP3	[33]
	Isoquinoline Alkaloids	Alkaloid	LPS-challenged broilers	Nrf2, TLR4/MyD88/NF-κB	[149]
	Viola yedoensis Makino	Plant Extract	LPS-challenged broilers	Nrf2/MAPK, NF-κB/NLRP3	[155]

### 4.4. Therapeutic Potential and Translational Outlook

The extensive body of evidence reviewed in this section establishes the Nrf2 signaling pathway as a validated therapeutic target for intestinal inflammatory disorders across multiple compound classes and experimental platforms. Across structurally diverse compound classes—ranging from phenolics and terpenoids to microbial metabolites—convergent protective mechanisms have been consistently observed, including antioxidant enzyme induction, NF-κB suppression, ferroptosis inhibition, and gut microbiota modulation. This mechanistic convergence underscores the fundamental role of Nrf2 signaling in maintaining intestinal homeostasis and highlights its therapeutic relevance across multiple disease contexts. The demonstrated efficacy in rodent disease models, livestock production systems, and human intestinal cell lines collectively establishes a robust preclinical foundation for clinical development, while concurrent agricultural applications present viable alternatives to antimicrobial growth promoters with attendant benefits for animal welfare and food safety.

Among the phenolic compounds, resveratrol and phenolic acids occupy a distinctive translational niche owing to their abundance in common dietary sources and well-established safety profiles. Their efficacy in weaned piglet models, which closely approximate human intestinal physiology, supports dual applications as livestock feed additives and nutraceutical interventions for inflammatory bowel disease management. Flavonoids, particularly quercetin and curcumin, exhibit exceptional translational potential through extensive validation across DSS-induced murine colitis, deoxynivalenol-challenged livestock, and human intestinal cell lines, with both compounds having advanced to human clinical trials as adjunctive therapies. Curcumin and epigallocatechin gallate represent the most clinically mature Nrf2 activators, demonstrating efficacy in multiple human ulcerative colitis trials while advanced delivery systems, including nanoparticles and liposomes, have successfully addressed historical bioavailability limitations. The emerging recognition of anti-ferroptotic mechanisms further expands their therapeutic scope to encompass chemotherapy-induced intestinal mucositis, thereby addressing a significant unmet clinical need.

Endogenous and dietary metabolites demonstrate similarly robust translational applicability. Amino acids and bioactive peptides, including glutamine, taurine, and betaine, offer immediate clinical relevance given their established utilization in clinical nutrition and animal feed formulations; the elucidation of Nrf2-mediated mechanisms now provides a rational basis for optimizing dosing regimens and developing targeted combination therapies. Lipid-based activators, particularly omega-3 polyunsaturated fatty acids, benefit from well-characterized dietary incorporation pathways and integrate intestinal barrier protection with established cardiovascular and metabolic benefits. Polysaccharides present exceptional advantages through their prebiotic properties and compatibility with existing dietary supplementation infrastructure, whereas organic acids currently employed as antimicrobial feed preservatives now possess mechanistic justification for their observed gut health benefits through Nrf2 pathway activation.

Microbiome-based interventions constitute particularly promising therapeutic strategies warranting focused development. Probiotics represent the most clinically advanced approach, with Nrf2 activation identified as a conserved protective mechanism across taxonomically diverse species, thereby providing a rational framework for strain selection and combination therapy design. The emergence of next-generation probiotics, including *Akkermansia muciniphila* and specific *Clostridium* species, opens additional therapeutic avenues. Microbial metabolites, particularly short-chain fatty acids and tryptophan derivatives, function as the mechanistic interface between dietary intake, microbiome composition, and host intestinal health. Butyrate supplementation is already employed clinically for ulcerative colitis, while postbiotic formulations comprising defined metabolite mixtures are under active investigation.

Plant-derived terpenoids and alkaloids benefit from detailed structural characterization that enables systematic structure-activity relationship optimization. Ginsenosides are progressing through Western clinical trials following extensive historical application in traditional Chinese medicine, establishing precedent for related terpenoid compounds. Berberine represents one of the most clinically advanced natural Nrf2 activators, with demonstrated efficacy in human metabolic and gastrointestinal disorders and regulatory approval in several countries for diarrheal diseases. Its established efficacy in enterotoxigenic *Escherichia coli*-infected piglet models further supports therapeutic development for both agricultural applications and pediatric gastroenteritis. Traditional herbal formulations offer the distinctive advantage of centuries of empirical clinical validation, and mechanistic elucidation of their Nrf2-dependent actions facilitates international regulatory acceptance while enabling the standardization essential for contemporary pharmaceutical development.

Future research priorities should include standardization of optimal dosing regimens across species, development of advanced delivery systems to overcome bioavailability constraints, and identification of patient subpopulations most likely to derive benefit from Nrf2-targeted interventions. Integration of omics-based biomarkers for monitoring pathway activation status will facilitate personalized therapeutic approaches and accelerate clinical translation of these promising natural therapeutics.

## 5. Conclusions

This review demonstrates that the Nrf2/Keap1 signaling pathway serves as a central therapeutic target for intestinal inflammatory disorders. Diverse agents—including polyphenols (resveratrol, quercetin, curcumin), terpenoids, alkaloids, polysaccharides, and probiotics—activate NRF2 through distinct mechanisms: direct Keap1 cysteine modification, upstream kinase signaling, and gut microbiota-derived metabolites. These compounds exhibit coordinated dual-pathway regulation, simultaneously enhancing Nrf2-mediated antioxidant defenses while suppressing NF-κB-driven inflammation. Beyond classical antioxidant induction, Nrf2 activation promotes multiple protective mechanisms including ferroptosis inhibition, tight junction reinforcement, and inflammasome suppression. The gut microbiota critically modulates therapeutic efficacy through metabolite production and compound biotransformation. These findings establish Nrf2 pathway modulation as a promising multi-targeted strategy for managing IBD in both clinical and livestock settings. However, several knowledge gaps and challenges remain. First, most studies rely on rodent models, and translational validation in larger animals and human clinical trials is needed. Second, the optimal dosing regimens, delivery systems, and long-term safety profiles of Nrf2 activators require further investigation. Third, the interplay between Nrf2 activation and gut microbiota composition warrants mechanistic studies. Future research should focus on developing species-specific formulations, investigating synergistic effects of combined Nrf2 activators, and conducting comparative efficacy studies across different livestock species.

## Data Availability

No new data were created or analyzed in this study.
